# INDIVIDUALS' KNOWLEDGE ABOUT A LOVED ONE’S END-OF-LIFE CARE WISHES IS ASSOCIATED WITH THEIR OWN ADVANCE CARE PLANNING

**DOI:** 10.1093/geroni/igac059.2526

**Published:** 2022-12-20

**Authors:** Peiyuan Zhang, John Cagle

**Affiliations:** University of Maryland Baltimore, Baltimore, Maryland, United States; University of Maryland, Baltimore, Baltimore, Maryland, United States

## Abstract

Despite evidence that ACP can improve quality of life for both patient and family, it remains underutilized. Only 37% US adults have a complete advance directive (Kuldeep et al., 2017). Existing studies primarily examined factors associated with patients’ ACP engagement but very few explored ACP among patients’ family. The goal of this study is to examine whether individuals’ knowledge about a loved one’s end-of-life (EOL) care preferences is associated with their ACP engagement. Data are from the US arm of the 2015 Four-Country Survey on Aging and End-of-Life Medical Care. The sample included N=609 adults who experienced the death of a family or close friend in the past 5 years.Three binary dependent variables were measured by different facets of ACP: having a serious conversation about EOL medical care wishes (1) with loved ones, (2) with doctors and (3) documenting those wishes. The primary independent variable was participants’ knowledge about their deceased family’s EOL treatment wishes.Three separate logistic regression models were used. Individuals who had a greater knowledge about their loved one’s EOL treatment wishes were twice as likely to have a conversation with family about their own EOL wishes (OR=2.32, P< 0.001) and documented wishes than those who didn’t(OR=2.03, P< 0.05). Results have direct implications for clinicians who work with families in EOL care settings. They may have opportunities to engage individuals – other than patients – in ACP. Focusing the experience of involvement in a loved one’s EOL care may be an effective strategy to enhance ACP engagement.

